# Gamification as a Tool for Understanding Mental Disorders in Nursing Students: Qualitative Study

**DOI:** 10.2196/71921

**Published:** 2025-06-20

**Authors:** Pablo Del Pozo-Herce, Alberto Tovar-Reinoso, Eva García Carpintero-Blas, Ana Casaux Huertas, Regina Ruiz de Viñaspre-Hernández, Antonio Martínez-Sabater, Elena Chover-Sierra, Marta Rodríguez-García, Raul Juarez-Vela

**Affiliations:** 1 Research Group on Innovation in Health Care and Nursing Education (INcUidE), UNIE University, Madrid, Spain; 2 Escuela de Enfermería Fundación Jiménez Díaz – Universidad Autónoma de Madrid (FJD-UAM), Campus de Villalba, Instituto de Investigación Sanitaria Fundación Jiménez Díaz (IIS-FJD, UAM), Madrid, Spain; 3 Madrid Renal Foundation, Madrid, Spain; 4 Research Group in Care GRUPAC, Department of Nursing, Faculty of Health Sciences, Universidad de La Rioja, Logroño, Spain; 5 Care Research Group (INCLIVA), Hospital Clínico Universitario de Valencia, Valencia, Spain; 6 Nursing Care and Education Research Group (GRIECE), Nursing Department, Universitat de València, Menendez y Pelayo, 19, Valencia, 46010, Spain, 34 639871179; 7 Internal Medicine Department, Consorcio Hospital General Universitario, Valencia, Spain

**Keywords:** gamification, mental health, nursing students, cognitive training, qualitative research

## Abstract

**Background:**

Gamification has emerged as an innovative pedagogical strategy in the educational field, transferring game tools to the teaching-learning process to improve students’ motivation and engagement.

**Objective:**

This study aims to describe nursing students’ perceptions of mental disorders using interactive cards as a gamification tool.

**Methods:**

This research was carried out at the Nursing School of a University in Madrid, Spain, with the participation of 50 first-year students enrolled in the nursing degree’s general and developmental psychology course. Data were collected through focus groups and reflective narratives with semistructured interview questions between March and April 2024. After data collection, transcripts were generated and subjected to thematic analysis following the Consolidated Criteria for Reporting Qualitative Research (COREQ) checklist.

**Results:**

A total of three themes emerged from the analysis: (1) perception and stigma of mental disorders, (2) emotional connection and personal reflection in learning about mental disorders, and (3) gamification tools and their impact on learning.

**Conclusion:**

Gamification, especially through interactive cards, is valuable for teaching psychology and mental disorders in nursing education. It enables students to gain a deeper clinical understanding of mental illnesses and explore their emotional and social dimensions. This methodology fosters emotional reflection, reduces stigma, and encourages active engagement, contributing to developing more empathetic, reflective, and better-prepared nursing professionals. Its integration into educational programs enhances academic and humanistic competencies essential for mental health care.

## Introduction

Over the past decade, gamification has emerged as an innovative educational strategy incorporating game dynamics, rules, and elements into non-game contexts, such as learning environments [1]. Its central aim is to transform traditional educational activities into interactive and engaging experiences, stimulating students’ intrinsic motivation and promoting active learning. Gamification introduces game—like components into the classroom to foster more dynamic and effective learning processes [2]. This approach is particularly effective when students are internally motivated, as it encourages deeper engagement with their educational journey [3]. It has shown notable success in complex fields such as health care, where integrating theory and practice is essential for developing competent and empathetic professionals [4]. In nursing education, gamification provides a dynamic and immersive framework through which students can engage with real-world challenges, enhancing their clinical, cognitive, and emotional competencies [5,6].

Recent research has highlighted the growing potential of gamification-based mental health interventions to improve emotional well-being and reduce psychological symptoms such as anxiety, stress, and depression [7,8]. These interventions use game design principles to increase users’ motivation, active participation, and engagement in their therapeutic processes. This innovative approach is not only transforming the way mental disorders are treated but also how their management is taught to health professionals. As an innovative strategy, gamification has applications in diverse educational contexts [5,6,9] and health interventions focused on people with mental health problems [8,10]. However, despite their advances and growing popularity, there is a notable gap in understanding how these gamified strategies impact both teaching and learning related to mental health.

One of the biggest challenges in nursing education is the understanding of mental disorders [11,12]. These pathologies require an in-depth knowledge of symptoms and an empathic ability to connect with patients’ personal and subjective experiences [13]. By implementing game dynamics, such as using interactive cards, students can explore, in a safe and structured way, the inner world of people with mental illness [14]. This methodology helps transform theoretical knowledge into meaningful learning experiences by putting the learner at the center of the process and allowing them to somehow “live” the realities of their future patients [15].

In the specific context of teaching about mental disorders, interactive cards representing various pathologies, symptoms, or clinical scenarios enable students to engage with realistic and meaningful experiences. These cards often include narratives or descriptive vignettes simulating the lived experiences of individuals with conditions such as depression, anxiety, schizophrenia, or bipolar disorder [16]. Through reading and reflection, nursing students are encouraged to recognize the clinical features of these disorders and consider their personal and emotional significance for those affected. This pedagogical strategy supports the development of empathic competencies and enhances students’ understanding of mental illness’s psychological and social dimensions [17].

In addition, gamification in this context facilitates the development of a comprehensive understanding of the patient [8]. Students can interact more actively and critically with the content by using dynamics such as interactive cards, encouraging deep analysis of the emotions, behaviors, and thoughts that characterize each pathology. This type of tool allows personalization of learning since each student can interpret situations from their perspective, enriching the process by exchanging ideas and experiences with peers [3].

In nursing education, it is essential to move beyond the acquisition of technical skills and to cultivate nontechnical competencies such as empathy, emotional regulation, and effective communication. These attributes are fundamental for delivering holistic and patient-centered care. Gamification serves not only to enhance students’ understanding of mental disorders but also to prepare them to engage with patients in an ethical, compassionate, and sensitive manner. Interactive cards, in particular, encourage future nurses to view patients as whole individuals—beyond their diagnoses—by considering their personal histories and social contexts. In this regard, gamified educational tools offer promising avenues for improving both the learning process and the depth of understanding related to mental health care.

This study aims to describe nursing students’ perceptions of mental health disorders using interactive cards as a gamification tool.

## Methods

### Design

An interpretive approach qualitative study [18] was conducted to investigate nursing students’ perceptions and experiences during an interactive card gamification activity focused on mental disorders. This paradigm describes how people construct their social reality and knowledge by reconstructing their experiences [19].

### Experience or Role of Researchers

A total of 9 investigators (4 women and 5 men) participated in this study, all with PhDs in health sciences and psychology, except for one nurse from the clinical setting with a specialty in mental health (PDP-H). A total of 4 investigators (EGC-B, AT-R, PDP-H, and ACH) had extensive experience conducting qualitative studies in health sciences. One of the investigators (PDP-H) was responsible for recruiting participants. The remaining authors had no prior contact with any of the student participants. The focus groups (FGs) were led by 2 researchers specializing in using gamification tools and active learning methodologies in mental disorders (PDP-H and AT-R). The researchers’ positions were established regarding the theoretical framework, beliefs, previous experience, and motivations for participating. The entire team participated in the evaluation of each stage of the research process to reduce researcher bias. Data were triangulated with 3 external researchers (AM-S, RJ-V, and EC-S).

### Participants and Sampling

This study was conducted at a university in the Community of Madrid, Spain, where the nursing degree spans 4 academic years. The course General and Developmental Psychology, part of the first-year curriculum, is a 6 European Credit Transfer System (ECTS) credit subject taught in the second semester. It includes both theoretical instruction and the development of subject-related competencies.

A purposive sampling strategy was used, selecting participants based on their ability to provide relevant insights aligned with the research questions [20]. Inclusion criteria were (1) first-year undergraduate nursing students and (2) an enrollment in the General and Developmental Psychology course at the participating university. Participation was voluntary and offered to all eligible students. Of the 53 students enrolled, 50 chose to participate (n=50). Recruitment continued until data saturation was reached [21], ensuring a comprehensive and information-rich sample.

All participants were first-year students with no formal mental health education; this course represented their initial academic exposure to related topics. While a few had previously experienced basic gamified activities during earlier stages of education, this study marked their first structured encounter with gamification as a pedagogical strategy within higher education.

### Teaching Strategy

**Figure 1.**
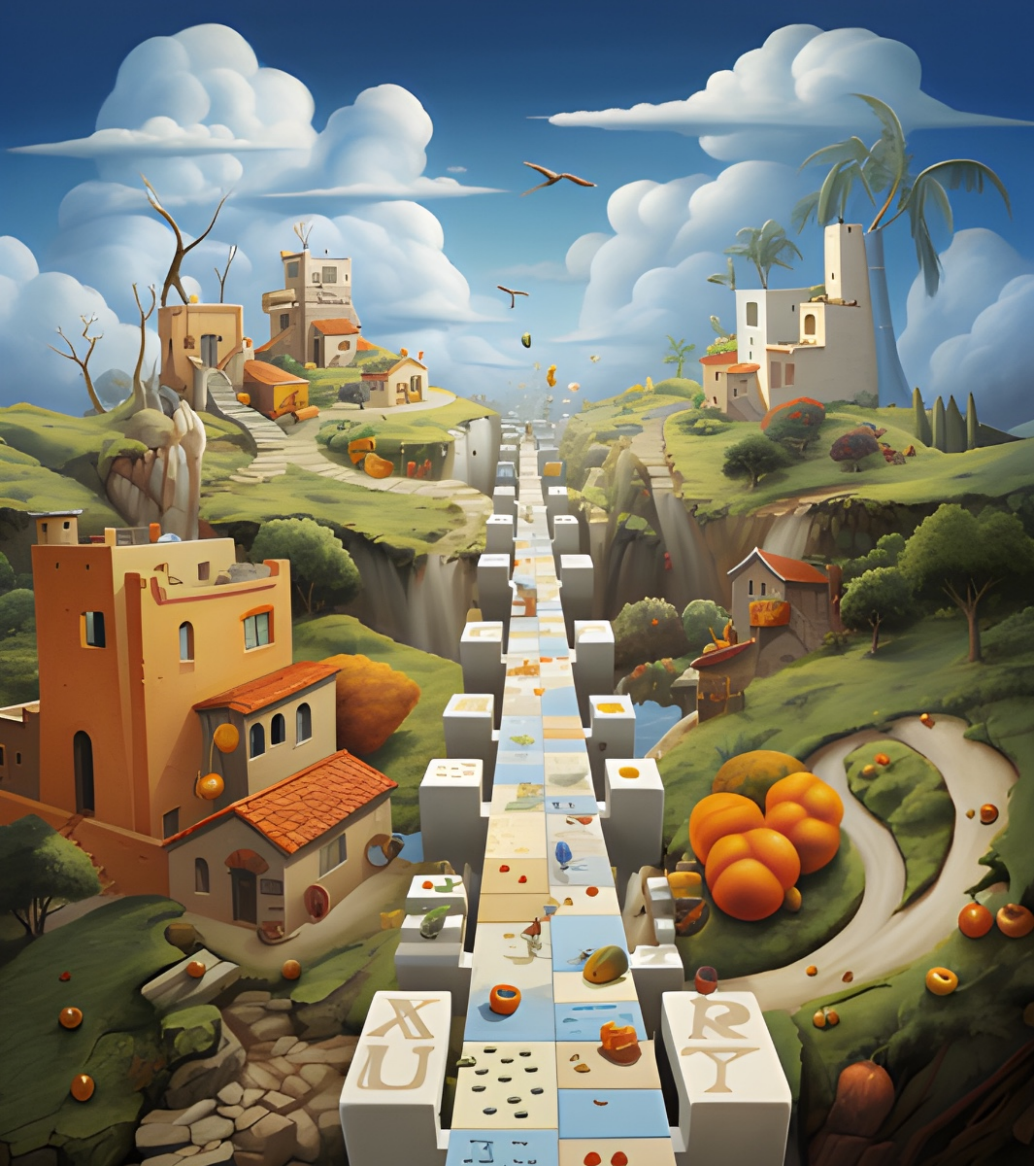
Interactive card 1.

As part of the educational intervention through gamification, a group activity specifically designed to explore perceptions about eating disorders (EDs), personality disorders (PDs), and emotional instability, such as anxiety and depression, was proposed to students. This intervention was implemented through interactive cards (see Figures1^4^), symbolic tools to facilitate reflection and self-awareness around these mental disorders. During the intervention, a fundamental distinction was introduced between conflict graphs and reframing graphs. These were directly linked to analyzing how students perceive and emotionally process the characteristics and challenges associated with mental disorders.

**Figure 2.**
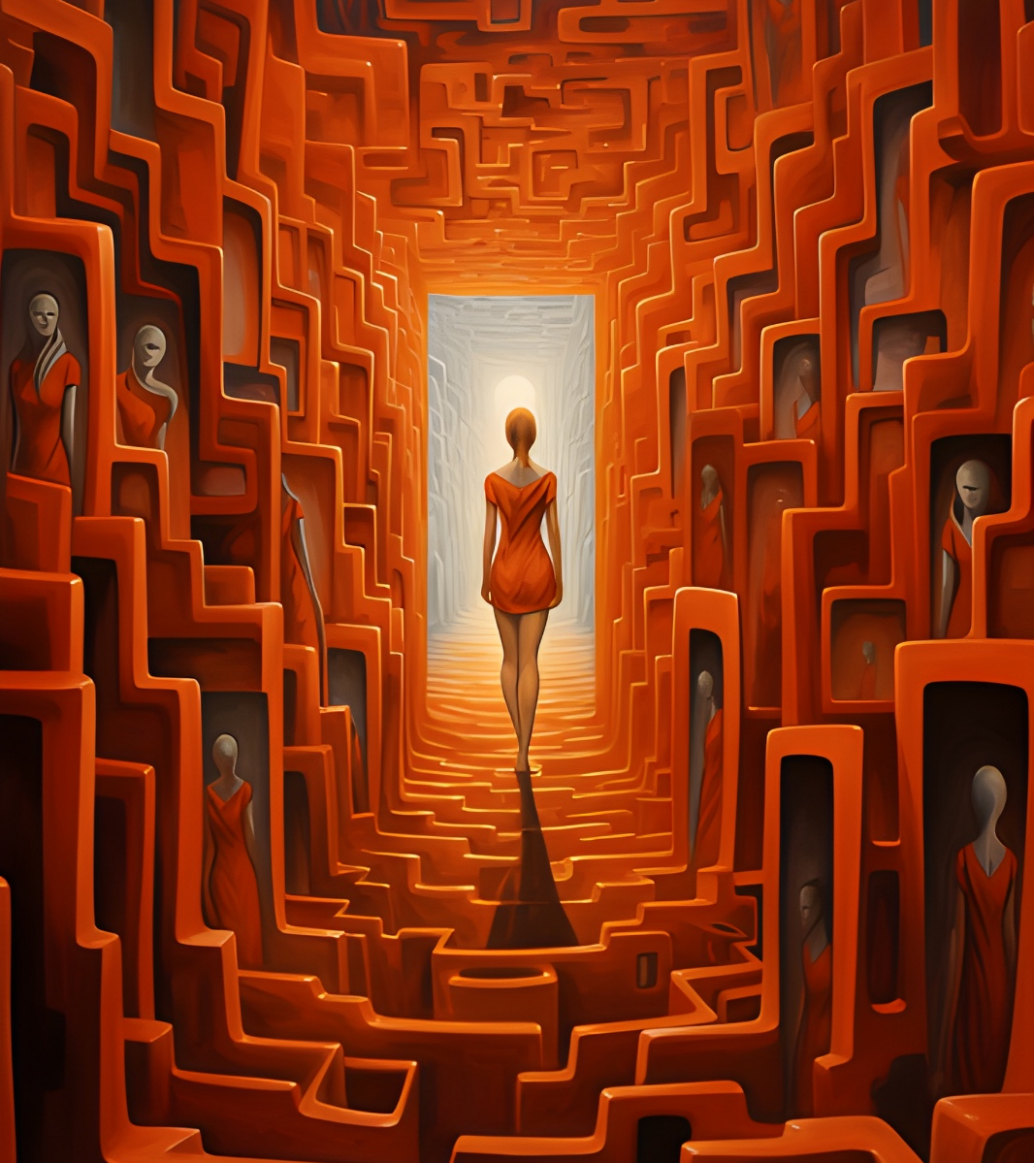
Interactive card 2.

**Figure 3.**
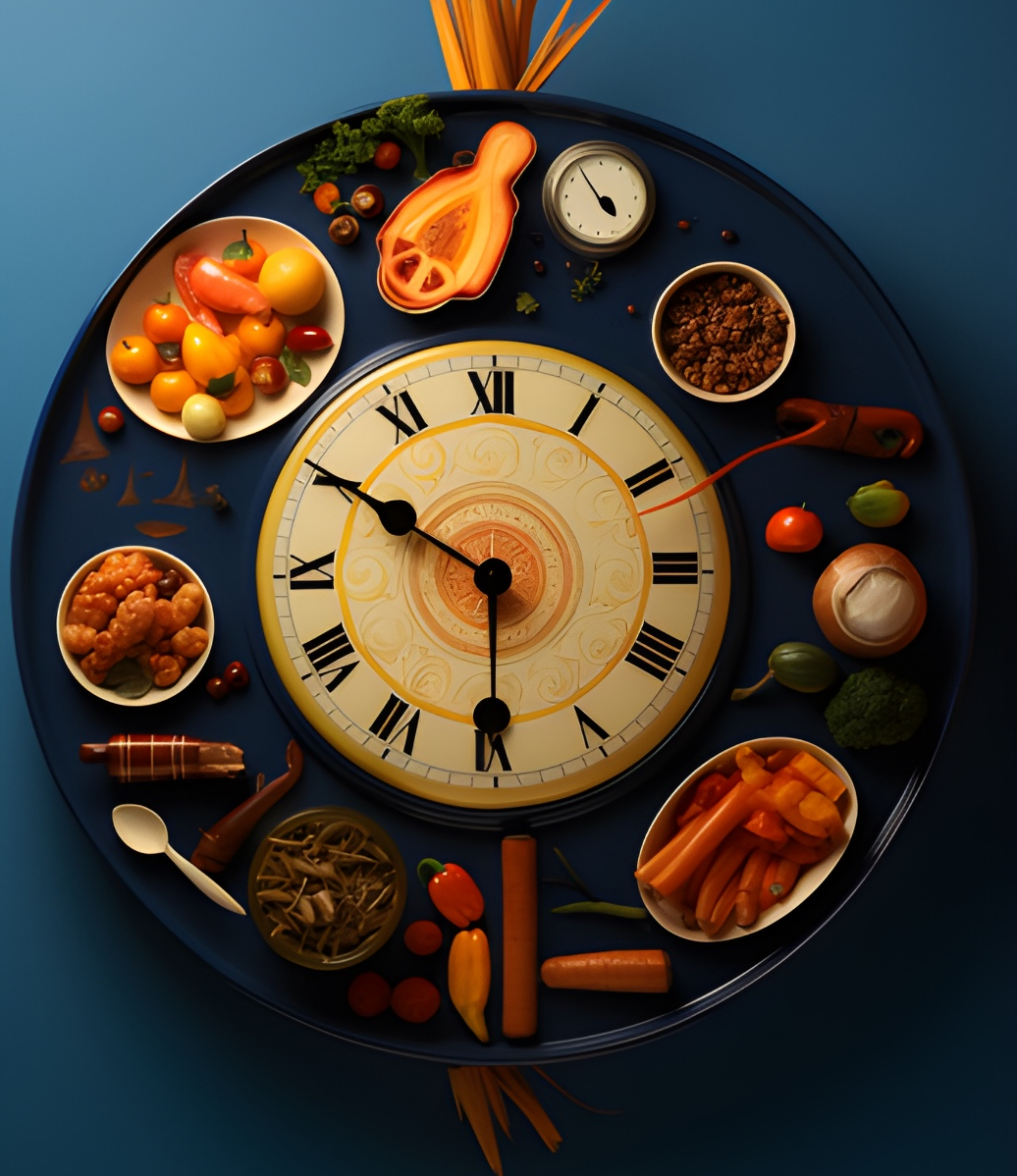
Interactive card 3.

**Figure 4.**
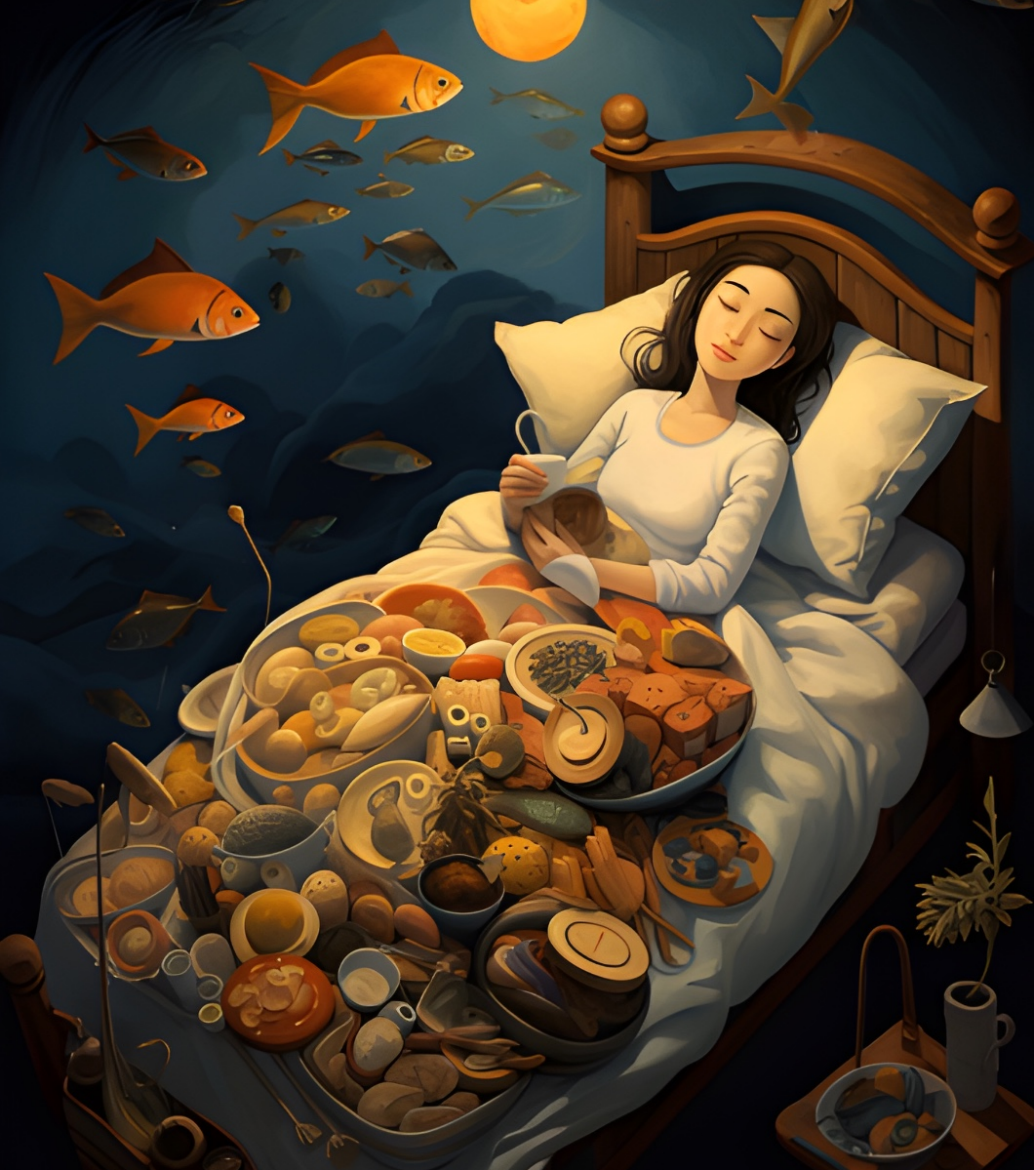
Interactive card 4.

Conflict cards represent emotional challenges, internal obstacles, or situations that generate distress, anxiety, or confusion, common elements in disorders such as ED and PD. Through these, students could visually identify problem areas in their emotional lives or associate them with experiences observed in people with mental disorders, fostering a deeper and more empathetic understanding. This process allowed them to reflect on how these internal conflicts might be related to the symptoms and challenges faced by those with mental disorders.

Reframing graphs symbolize a new perspective or emotional solution, providing learners with an optimistic view or path to resolution similar to the therapeutic goals of treatments for mental disorders. This exercise encouraged participants to project how to overcome the challenges represented by the conflict graphs, visualizing possible ways to cope with and manage anxiety, emotional instability, or challenges specific to ED and PD.

The interactive cards functioned as emotional metaphors, enabling students to explore their internal experiences in a tangible and visually expressive manner. This method allowed them to contextualize personal reflections within the broader framework of symptoms and lived experiences associated with various mental health conditions. Each student constructed the symbolic meaning of the cards individually, fostering a deeply introspective and subjective approach to emotional analysis. This personalized engagement supported the study’s objective of enhancing students’ understanding of and sensitivity toward mental disorders’ complexities (see Multimedia Appendix 1).

The activity consisted of each group preparing a document that included the following elements:

First, the association of interactive cards with mental disorders; the students were asked to identify and justify which interactive card was associated with each of the disorders presented: eating disorders, personality disorders, anxiety, or depression, justifying this association. This task was intended to promote individual and collective reflection, encouraging participants to explore the symbolic relationship between the interactive cards and the disorders or emotions represented. This phase aimed to get students to deepen their symbolic analysis of the interactive cards and connect the images to their knowledge of mental disorders. The exercise encouraged group discussion, generating different interpretations and a shared understanding of the disorders from an emotional and visual perspective.

Second, the analysis of the abstraction and meaning of the interactive cards was conducted. After addressing the type of disorder and its impact on those who are diagnosed with it, we delved into introspective aspects of the students, such as whether they have ever felt similarly, their fears, problems, and their ideal or desired state. They also reflected on how they believe people with ED experience it and how they would feel if they experienced it personally or if someone close to them had gone through this situation.

The central objective of the gamification activity was to train students in understanding and managing mental disorders through interactive card-based experiences that promoted reflection and emotional analysis. This approach allowed students to develop nursing-specific competencies to address mental health-related situations effectively. For this purpose, a variety of interactive cards representing different mental disorders, such as eating disorders, anxiety, and depression, were used.

Before using the interactive cards, consent was requested from their creator, Alejandro Vera Casas, who approved their use and is knowledgeable about the study and intervention with the students.

### Data Collection

To explore diverse viewpoints, FGs were convened along with the researchers’ field notes and participants’ written reflective narratives, which enriched the analysis. This qualitative methodology allowed for a nuanced and contextualized exploration of lived experiences during the interactive card-based gamification activity on mental disorders [22]. The FGs encouraged interaction among participants, fostering the emergence of varied opinions and perceptions regarding their feelings and learnings [20]. Data collection was conducted between March and April 2024, facilitating a detailed topic analysis [23].

Each group was composed of between 9 and 11 participants, guided by a moderator and accompanied by an observer. The FGs were developed at the university after the gamification activity, with participants seated and facing each other to encourage interaction, data collection, and direct observation. The moderator asked questions, and each participant responded in an orderly manner. The observer complemented the moderator’s work, highlighting key points and taking notes. A thematic guide was used, which, although focused on obtaining information specific to the study area, allowed sufficient flexibility to promote discussion and interaction among participants (see Textbox 1). Due to the flexible nature of data collection in qualitative studies, the moderator explored additional themes that emerged from the participants’ interventions and related to the research question. All FGs were audio-recorded with the participants’ prior permission. A total of 5 FGs were conducted in which no new information emerged from the data analysis, with an average duration of 79 minutes. Before analysis, participants had the opportunity to review the transcripts. In addition, researchers’ field notes were used as a secondary source of information.

Textbox 1. **Semistructured interview questions.**

1. How did you feel about the development of using interactive cards?
2. What has it helped you to relate to mental disorders or learning?
3. What has meant using interactive cards as a gamification tool for your learning?
4. How do you think you could use what you have learned in your professional future?
5. How have interactive cards helped you to integrate content?
6. Has it made it easier to understand?
7. Have you encountered any difficulties while doing the activity?
8. Would you feel comfortable doing the activity in a group or prefer to do it individually? Why?

The results were also triangulated with the 32 individual narratives written by the participants, in which they shared their experiences of managing mental disorders through interactive cards. These narratives included personal reflections and an in-depth analysis of their emotions.

### Data Analysis

Verbatim transcriptions were made for each FG alongside researchers’ field notes and reflective narratives. All data were carefully stored, managed, and organized using ATLAS.ti (version 24; Scientific Software Development GmbH), a qualitative data analysis software. An inductive thematic analysis [18] was conducted to identify relevant text segments addressing the research question systematically. The analysis began with open coding of the transcripts, during which researchers identified and labeled meaningful units of text. These initial codes were then grouped into categories based on shared meanings and thematic similarities. The categories were refined and organized into broader thematic groups through an iterative process of constant comparison. This interactive and reflective approach ultimately identified overarching themes that captured participants’ experiences, offering a rich and nuanced understanding of their perceptions [24].

A total of 4 researchers (EGC-B, AT-R, PDP-H, and ACH), experts in qualitative research, developed the whole process of obtaining categories and subcategories independently, ending the process with the exchange of both and a consensus on the final decisions of the analysis. In case of divergence of opinions, the theme was identified by consensus among the research team members. In addition, memo writing and analytic discussions were used throughout the process to support reflexivity and maintain analytical rigor.

### Rigor and Trustworthiness

The study followed the Consolidated Criteria for Qualitative Research Reporting (COREQ) [25]. The criteria to ensure the reliability of Guba and Lincoln were applied [21]. Data triangulation was used among the researchers involved in the analysis, and the analysis process was subjected to independent researcher review to ensure credibility. Transcripts were offered to participants, who were allowed to add any relevant information. Transferability was ensured by a detailed description of the research setting, participants, context, and method. Confirmability was achieved by introducing variability in participants’ experiences. Each researcher conducted the reading and analysis independently, contrasting and agreeing on emerging themes and subthemes.

### Ethical Considerations

The study was submitted to the Research Ethics Committee of the Instituto de Investigación Sanitaria Fundación Jiménez Díaz (CEIm-FJD) for review. Although the committee determined that the project fell outside its scope as it did not constitute biomedical research, it found no ethical objections to the documentation provided. All participants gave written consent before participating in this study. To ensure anonymity and confidentiality, a code was assigned to each participant in the FGs and reflective narratives. Participants were recruited through an open call disseminated through institutional channels, including emails and posters. They were informed in detail about the study, its objectives, and procedures, emphasizing that participating or not would not affect their academic career. It was made clear that participation in the study was entirely voluntary, would have no impact on their grades or academic evaluation, and they could withdraw at any time without any consequences on their academic performance or relationship with the university. Strict measures were implemented to ensure neutrality between teaching and research: data collection and interventions were anonymous and did not alter course dynamics, while academic results were managed independently. These actions fully protected students’ learning rights.

## Results

### Participant Characteristics

Of the 53 nursing students who met the inclusion criteria, only 50 participated in the study, representing a participation rate of 94.3% (50/53). A total of 3 students did not attend the gamification activity due to scheduling conflicts, work commitments, or other personal reasons. Most participants were female (44/50, 88%), while 6/50 (12%) were male. The average age of participants was 18.5 years (SD 1.28; range 17-23 years). The gender imbalance reflects broader enrollment trends in nursing programs across Spanish universities, where female students constitute the majority. All participants were first-year nursing students enrolled in the General and Developmental Psychology course.

### Themes

From the interactions developed during the gamification activity, three thematic blocks were identified along with their respective categories (see Table 1): (1) Perception and stigma of mental disorders; (2) Emotional connection and personal reflection in learning about mental disorders; and (3) Gamification tools and their impact on learning.

**Table 1.** Thematic analysis overview: themes, categories, and subcategories.

Themes (T)		Categories	Subcategories
T1: Perception and stigma of mental disorders		Beliefs Stereotypes and prejudices Understanding level Perception of interactive cards	Myths and initial beliefs Stigmas in the social environment, unconscious prejudices, and awareness of the impact Differentiation between disorders, understanding of severity, and identification of symptoms Emotional impact, learning transformation, open mind, usefulness, and difficulties encountered
T2: Emotional connection and personal reflection in learning about mental disorders		Personal and social environment Accompaniment and support networks Role of mental health professional Attitudes change	Influence of family expectations and close environment Family support, barriers, and support network Intervention strategies, therapeutic relationship, and more integral vision Awareness and sensitization
T3: Gamification tools and their impact on learning		Development of emotional and empathic skills Learning process assessment Introspection and self-knowledge Impact on empathy development	Improved emotional communication skills to identify emotions Better understanding, meaningful learning, and integration of knowledge Personal reflection, identification of experiences and biases, and insecurity Recognition of other people’s emotions, desire to help, and emotional connection.

#### Theme 1: Perception and Stigma of Mental Disorders

##### Beliefs

This category explores initial myths and beliefs and changes in students’ perception of mental disorders through their interaction with visual material, such as the interactive cards used in the activity. They highlight the pedagogical value of the images and interactive cards in understanding mental disorders more deeply.

*In future courses, these cards can be employed as a resource to associate the mental pathologies we study.*

[FG 1]

When confronted with these resources, self-awareness has been awakened in them that has not only allowed them to rethink their beliefs about mental disorders but also their emotional well-being:

*I have questioned my own experiences.*

[Participant 15]

##### Stereotypes and Prejudices

Through the activity, participants reflected on how these stigmas affect both how society perceives people with mental disorders and how these people see themselves. The reflections reveal a change in understanding and an increased awareness of the impact of prejudice on the lives of those affected. They have been able to reflect on the role that erroneous beliefs, unconscious biases, and social expectations play in how these people see themselves and how they feel judged by others.

*We thought it may be a case of personality disorder due in large part to prejudice and lack of confidence*.

[FG 5, related to interactive card 1]

Through the interactive cards, the students observed that certain mental disorders, especially the most common ones, tend to be more stigmatized. This recognition allowed them to analyze how social stigma is disproportionately associated with certain disorders, which hinders access to help and adversely affects the well-being of those affected.

*In the* interactive cards, *they observe the different types of disorders that are more stigmatized and more common*.

[Participant 46]

The free approach to the activity allowed them to deepen their understanding of mental disorders without being constrained by preconceived ideas, which contributed to a more open reflection on the associated prejudices and stigmas.

*Being a flexible and free-viewing activity, it helps to think about how a person suffering from a disorder might feel.*

[Participant 12]

##### Understanding Level

Student reflections revealed varying levels of understanding, ranging from the recognition of symptoms to a deeper internalization of the emotions and thoughts associated with different mental disorders. Participants demonstrated the ability to distinguish among various conditions, a crucial step toward comprehending the unique severity and impact. As a student explained,

*In the image, we see a connection between time and food… we were able to associate different foods with each hour of the day, and it is reflected with an obsession with scheduling, meticulous planning of food intake*.

[Participant 16]

This differentiation process enabled students to recognize specific symptoms and interpret how these disorders deeply affect the emotional and cognitive experiences of those who live with them. As one participant reflected,

*Each time it tends to infinity; that infinity is like associating it with something chronic—of an eating disorder*.

[Participant 10]

Such reflections reveal a growing capacity for nuanced understanding that extends beyond surface-level identification, indicating the development of empathetic insight and clinically relevant perspectives.

As students progressed through the activity, they demonstrated an increasingly sophisticated understanding of the varying levels of severity associated with mental disorders—ranging from observable behaviors to more complex emotional and psychological dimensions. They moved beyond simply associating symptoms and behaviors, gaining insight into the emotional toll experienced by individuals living with these conditions, including struggles with control and the anxiety tied to rigid eating patterns. This multidimensional understanding highlights how interactive cards support theoretical learning and foster empathy and emotional connection with the lived reality of mental illness. As a student reflected,

*Maybe she is aware that she is in that darkness, but she doesn’t want to get out because even though she feels bad about herself, she doesn’t feel bad—she’s comfortable, she doesn’t want to get out of there*...

[Participant 2]

##### Perception of Interactive Cards

The participants’ perceptions highlight the value of using interactive cards as an educational tool in the study of psychology, particularly in enhancing understanding of mental disorders. The activity was widely appreciated for transforming theoretical content into a more accessible, engaging visual and interactive experience. A participant explained,

*I found this way of integrating knowledge easier than a more theoretical one. When you see it in an image and have to reflect on its contents, you have to associate all the information you have given us in class to be able to put it into practice.*

[Participant 22]

In addition, most participants emphasized the benefits of working in groups, noting that exchanging diverse interpretations and perspectives enriched the learning experience. Group interaction fostered an atmosphere of trust and respect, encouraging deeper reflection and more balanced conclusions. As one student stated,

*We found it more comfortable to carry out the activity in a group because we were able to deepen our reflection on each interactive card since we had more than one point of view*.

[Participant 16]

While participants acknowledged the benefits of the activity, some also highlighted specific challenges—particularly related to the abstract nature of the images, which required considerable interpretative effort. The process of identifying and associating symbolic elements with specific mental disorders was perceived as complex, necessitating an open-minded, analytical, and reflective approach. A student observed,

*I find it difficult to associate the interactive cards with a disease. I think it is complicated to diagnose, and maybe the signs given by the image have nothing to do with what the author of the photographs really intended*.

[Participant 21]

Despite these difficulties, participants recognized the pedagogical value of engaging with ambiguity, noting that it mirrored the interpretive and diagnostic complexities often faced in real clinical settings. The experience thus served as an essential formative exercise, encouraging the development of critical thinking and diagnostic sensitivity. Furthermore, the activity helped to strengthen their connection to the field of psychology, providing early exposure to its multifaceted nature and emphasizing its relevance to their future roles as health care professionals.

### Theme 2: Emotional Connection and Personal Reflection in Learning About Mental Disorders

#### Personal and Social Environment

This category illustrates how personal and social environments significantly influence the emotional and mental experiences of individuals, particularly those living with mental disorders. When engaging with the interactive cards, participants frequently highlighted the distress and anxiety associated with being judged by others and how such judgments shape their self-perception and social behavior. An FG participant reflected,

*We have felt very distressed and overwhelmed by how you are judged... It seems like a tunnel that looks like a labyrinth... when you are judged, the only thing you do is get lost in your thoughts... generating anxiety.*

[FG 2, related to interactive card 1]

Participants also emphasized the powerful impact of societal expectations on self-image. Their feelings of inadequacy or failure to meet imposed standards influence how individuals view themselves and present themselves in social contexts. The metaphor of “mirrors” represented the weight of external expectations, underscoring how individuals may feel lost or disconnected when constantly comparing themselves to idealized social norms. As a participant stated,

*We interpret that the person may find themselves at a loss or judge by others’ expectations... This influences the way he/she shows him/herself to society.*

[Participant 38]

These insights underscore the importance of understanding the relational and societal dimensions of mental health, highlighting how external judgment and social pressure can exacerbate emotional distress and contribute to the internal struggles faced by those with mental disorders.

Other participants highlighted the influence of society in the understanding of mental illnesses, recognizing how these pathologies affect not only the people who are affected from them but also their close environment.

*We believe that interactive cards have allowed us to understand how mental illness affects people and how it can have an impact on the environment, the society.*

[FG 4]

Through various interpretations, the effects of mental health in the social context are valued, highlighting the importance of understanding the connections between self-image, food, and social perceptions.

#### Accompaniment and Support Networks

Participants reflect on the importance of accompaniment and support networks in the context of mental disorders. They highlight the challenges faced by people who lack family or social support and the crucial role of these networks in the recovery process. They acknowledge that the recovery process is not linear and that relapses may occur. However, they stress that, despite difficult moments, accompaniment and support can help the person to continue on the road to recovery:

*You can fall because you are not in a good moment... there can be relapses, but in the end, everything leads to the same place, which follows the path*.

[Participant 9, related to interactive card 2]

A central theme in this category is the lack of support faced by many people with mental disorders. Several participants pointed out that those without support networks, whether family or social, tend to experience more difficulties in their recovery process. Lack of resources and isolation can negatively affect self-esteem and self-image, making it challenging to follow appropriate treatment.

*She knows that what surrounds her is darkness; many people who have a mental pathology have no family, no resources, no means, in the end, that has an impact on the follow-up and her own self-esteem and self-image.*

[Participant 26]

Participants also reflect on the obstacles and barriers in readapting to a healthy routine, identifying relapses as moments in which external support is essential.

*In addition, we consider that houses dirt roads could represent the ’stops, relapses or downs’ of the process to readapt to a stable and healthy routine of meals*.

[Participant 8, related to interactive card 2]

#### Role of Mental Health Professional

Students reflected on the role they will assume as future mental health professionals, emphasizing the integration of the knowledge acquired during their training to enhance care for individuals with mental disorders. They identified their understanding of eating disorders, personality disorders, and trauma as essential foundations for their future clinical practice. As a participant expressed,

*In our professional future, we could use this knowledge to address similar cases in the field of psychology... integrating the acquired knowledge of eating, personality disorders, and addressing trauma into our professional practice*.

[Participant 48]

The 2 central pillars frequently mentioned as critical to effective intervention were: establishing a strong therapeutic alliance and implementing evidence-based strategies. Participants acknowledged the need to combine empathy with scientifically supported methodologies to promote patients’ emotional well-being and recovery.

*This would include implementing evidence-based intervention strategies and establishing a strong therapeutic relationship to promote my patients’ emotional well-being and recovery*.

[Participant 15]

Furthermore, students highlighted the value of adopting a holistic perspective in mental health care. Rather than focusing solely on visible symptoms or first impressions, they advocated for a more comprehensive approach that considers emotional, social, and psychological dimensions in an integrated manner. As stated in one FG,

*Not to be so rigid with opinions and try to see beyond what we can appreciate at a glance, that is, not to stay only with the initial but to devote time and effort to see everything in a more holistic way*.

[FG 1]

These reflections demonstrate the development of a professional identity rooted in empathy, critical thinking, and a commitment to evidence-based, person-centered care.

#### Attitudes Change

Participants reported a shift in their attitudes toward greater empathy, particularly as they began to understand the anxiety and distress caused by the uncertainty surrounding recovery from EDs. The lack of a clear resolution to the struggle often leads to heightened emotional distress for affected individuals. This insight made students more aware of the profound emotional burden these disorders entail. As an FG participant reflected,

*What we interpret from this image is that it represents a person who has hit rock bottom... the weight that food generates in his mind causes him nightmares*.

[FG 3, related to interactive card 3]

These reflections contributed to a notable change in how students perceive individuals with EDs, fostering a recognition of the diversity and complexity of their experiences and the importance of a nuanced and empathetic understanding. Several participants also expressed a deepened appreciation for the severity of EDs and other mental health conditions, with some indicating a desire to explore the topic further to support those affected better. A student shared,

*I have felt overwhelmed in general with the four interactive cards, as it considers mental pathologies as something serious and difficult, and I have to integrate myself into the subject to try to understand it*.

[Participant 36]

Through reflection and analysis of the lived experiences represented in the interactive cards, students developed a more empathetic and comprehensive perspective on the ongoing struggles and emotional challenges faced by individuals with mental disorders. This attitudinal shift underscores the value of sensitizing future health care professionals to the seriousness of mental illness and promoting an informed, compassionate approach to understanding and care.

### Theme 3: Gamification Tools and Their Impact on Learning

#### Impact on Empathy Development

Students’ perceptions suggest that the activity functioned as a meaningful catalyst for deepening their understanding of mental disorders and enhancing their capacity for emotional recognition and connection with affected individuals. Many participants described experiencing a strong sense of identification through empathically placing themselves in the position of those living with such conditions. As an FG participant expressed, this involved, “putting themselves in the shoes of people suffering from different disorders, having identified eating disorders, anxiety, depression, and personality disorder” (FG 3).

Testimonials also revealed a profound sense of empathy and compassion, particularly when engaging with the images used in the activity. This emotional response often translated into a genuine desire to offer support:

*"I feel empathy and compassion when viewing the images; it generates in me a desire to help and support those suffering from eating disorders, depression, and anxiety*.

[Participant 12]

Such responses suggest that the activity encouraged emotional resonance and identification with the challenges faced by others, but also inspired a meaningful commitment to action.

#### Introspection and Self-Knowledge

The results reveal that the activity has been a significant means of personal reflection for the students. The tool used has allowed them to express their emotions and share experiences in an intimate and personal way. On the one hand, it has helped them to reflect on themselves, highlighting the importance of self-knowledge in their emotional process:

*They allow us to express our emotions and to pour ourselves out in a personal way.*

[FG 2]

*Besides that, it has helped us to reflect on ourselves.*

[FG 3]

A participant mentioned that he identified with insecurities that he had experienced throughout his life, which highlights the relationship between the activities and the personal experiences of each individual:

*I find the interactive cards overwhelming as well as reflective, and they have caused me anguish, concern or even physical discomfort, since in some I have felt identified with insecurities that have arisen throughout my life.*

[Participant 8]

This suggests that the activity not only allows the expression of emotions but also invites them to confront and identify their personal experiences and biases. Likewise, the activity promoted reflection on the mental and emotional health of some participants, leading them to question their personal experiences:

*The interactive cards generate intense emotions in me such as sadness, fear or discomfort, recalling my own struggles and challenges.*

[Participant 12]

*It helps to express feelings, emotions that we do not know how to explain and shape, such as anxiety.*

[FG 1]

These findings suggest that the process of inspection and self-knowledge generated through the activity constitutes a valuable resource for emotional development.

#### Learning Process Assessment

They highlight the effectiveness of the activity in broadening the understanding of mental disorders and fostering critical reflection on them. Several noted that the activity allowed them to “broaden the mind and see beyond what is depicted on the interactive cards” (FG 5), promoting deep inquiry and reflection on the various layers of meaning in the images. They recognized that an image does not have a single meaning and that, like mental disorders, perceptions can vary significantly depending on the point of view. This experience helped them challenge the idea that a person with a mental disorder can be pigeonholed into a single experience or interpretation.

*They allowed me to understand that an image does not have to have only one meaning...a single thing can represent a great variety of sensations and impressions depending on one’s point of view.*

[Participant 12]

It emphasizes that each representation can evoke a variety of sensations and impressions depending on the observer’s viewpoint. Testimonials also reflect the importance of approaching sensitive topics with care and respect. A participant emphasized the ability to “draw conclusions with a careful and thoughtful approach” (P27)*,* suggesting that the activity focuses on theoretical understanding and the ethics of communicating about sensitive topics. This critical reflection is crucial for developing respectful and conscientious professional practice in the mental health field.

Likewise, it was mentioned that the activity is an excellent way to integrate the knowledge acquired in class:

*...good activity to integrate the knowledge obtained in class, adding the sensations that each disorder can arouse in the viewer and therefore further ingraining the knowledge of the various disorders.*

[FG 1]

Integrating theoretical knowledge with practical, emotional experiences reinforces the understanding of the various disorders and facilitates a deeper connection with the material studied. Finally, the interactive cards’ interpretation helped participants gain a “graphic view of the different mental disorders” (Participant 32) and the topics covered in the course, which translates into more meaningful and visual learning. They suggest the activity is a valuable resource to enhance learning and foster a more holistic and empathetic understanding of mental disorders.

#### Development of Emotional and Empathic Skills

Students reflected on how the activity significantly contributed to developing emotional skills for addressing mental disorders. Interactive cards were seen as an educational tool and a potential aid in clinical settings, facilitating patient openness and expression. An FG participant noted:

*Through the interactive cards, the patient can become more open and share deeper aspects of their personal experience, such as when asked to draw themselves.*

[FG 1]

The value of creative dynamics in therapeutic processes was emphasized, particularly in enabling patients to express experiences and emotions that may be difficult to articulate verbally. Furthermore, participants recognized the potential of these tools for early detection and assessment:

*The interactive cards can be valuable tools for early detection of mental health problems and for assessing the emotional state of patients*.

[FG 2]

These reflections underscore the broader applicability of the activity, not only in education but also in clinical practice and mental health research.

This perspective underscores the value of interactive cards within individual clinical contexts and as valuable tools in broader educational and research initiatives to deepen the understanding of mental disorders. Participants emphasized adopting a biopsychosocial framework when analyzing the interactive cards. This approach involves dedicating time and effort to constructing shared meanings beyond superficial interpretations. As an FG participant noted, it is vital "not to stay only with the initial but to dedicate time and effort to see everything in a more holistic and integrated way with a common meaning elaborated (FG 4).

Such an integrated perspective is essential for developing critical competencies in psychology. Encouraging students to consider emotional, psychological, and social dimensions together fosters a more comprehensive and empathetic understanding of patients’ lived experiences. Ultimately, it cultivates reflective, thoughtful, and patient-centered mental health professionals.

**Figure 5.**
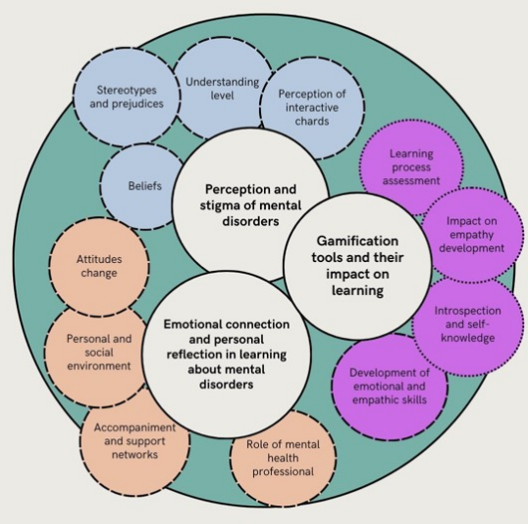
Qualitative data analysis.

These results reflect the complexity of understanding mental disorders through gamification as a learning tool. Figure 5 allows us to establish the following themes: the first theme deals with the perception and stigma of mental disorders. The second theme deals with emotional connection and personal reflection in learning about mental disorders. The third theme deals with gamification tools and their impact on learning. All of them are interrelated with each other through the ATLAS-TI program.

## Discussion

### Principal Findings

This study explored nursing students’ perceptions of the approach and management of mental disorders through gamification experiences, using interactive cards as an educational tool. Gamification is presented as an innovative pedagogical approach that transforms the learning process, providing students with an active and participatory experience that encourages critical reflection and meaningful learning [1,3,26,27]. Results suggest that using interactive cards not only stimulates students’ critical reflection on mental disorders but also allows them to challenge their theoretical knowledge, thus promoting growth in their self-confidence and ability to cope with complex situations in the mental health field [28-30].

These findings can be interpreted within the experiential learning framework, as the gamified activity immerses students in realistic scenarios that demand active decision-making, emotional involvement, and reflection. By confronting stigmatizing beliefs and exploring different perspectives through role-play and interaction, students engage in a process that aligns with transformative learning theory, fostering a change in their mental models and attitudes toward individuals with mental health disorders [31]. In this way, gamification serves as a motivational tool and becomes a vehicle for deep personal and professional growth [26-29].

Currently, gamification is gaining ground in clinical practice as an innovative strategy in the treatment and prevention of certain mental disorders. Several studies have shown that gamified interventions can improve patient engagement and motivation, facilitating treatment adherence in disorders such as depression and anxiety [7,8,32]. These tools allow patients to perform self-care, emotional regulation, and coping skills exercises in an interactive way, which can reduce symptoms and improve overall well-being. In addition, gamification in mental health can be tailored to each patient’s needs, personalizing the experience and promoting active, hands-on learning of life skills [8,32]. However, despite the proven benefits of mental health, gamification is still being implemented progressively in the academic training of health sciences students. These benefits highlight the importance of the intervention carried out in the present research, which addresses and studies the treatment of mental health disorders such as eating disorders, emotional instability, and personality disorders from a gamified approach.

Implementing these strategies in the classroom could facilitate the understanding and managing of these mental illnesses, promoting the development of practical skills and greater empathy in future nursing professionals. Currently, nursing students require alternative and innovative methods to maintain a high level of engagement in their learning process. Gamification has demonstrated significant benefits in the development of cross-cutting clinical competencies, favoring essential qualities such as resilience, confidence in teamwork, effective communication, resource management, and taking responsibility in collaborative roles [3,27]. These innovative approaches promote more dynamic learning and reinforce key skills needed in the clinical setting, with encouraging results [28,33]. Furthermore, gamification, by using game dynamics in educational contexts, motivates students to engage more deeply with the study material [3,26,34].

The research findings highlight that gamification, by incorporating emotional and realistic elements, maximizes the positive impact on students’ professional and personal development [29]. Students’ interest was stimulated by interactive cards representing different mental disorders. They could participate through FGs in in-depth and diverse discussions, which enriched their understanding of the topic and helped them develop essential interpersonal skills and emotional intelligence [3]. These skills include empathy, active listening, and effective communication, which are fundamental to establishing therapeutic relationships with patients [15,30,35].

Through the collaborative exploration of interactive cards, students were able to identify and critically analyze the signs and symptoms associated with eating disorders and emotional instability. This process facilitated the recognition of clinical manifestations and deepened their understanding of the emotional and social contexts that shape each mental health condition. By engaging with the complexities of mental disorders from multiple perspectives, students developed a more nuanced and empathetic appreciation of the lived experiences of individuals affected by these conditions [36].

Participants emphasized the importance of understanding the underlying motivations behind the behaviors of people with mental disorders. This reflects a more holistic and balanced approach that moves beyond a narrow focus on risk factors to acknowledge the role of protective factors. Such insights support the development of a comprehensive perspective on mental health, reinforcing the need for multidimensional assessments that integrate emotional, social, and clinical considerations in practice [36].

This level of insight is fostered through educational strategies that immerse students in emotionally meaningful and reflective learning experiences, aligning with the principles of experiential learning, as students are immersed in simulated, meaningful scenarios that demand reflection, analysis, and decision-making. Furthermore, the activity design fostered dialogical and collaborative learning spaces—through FGs and discussions—that facilitated the development of interpersonal skills such as empathy, active listening, and therapeutic communication [31]. Taken together, these processes also resonate with the principles of transformative education, as the students not only engaged in active, experience-based learning but also reported having questioned their preconceived ideas and developed a more nuanced and compassionate understanding of mental illness.

One of the key advantages of gamification lies in the positive perceptions students hold toward educational games, which enhance motivation and promote learning across cognitive and affective domains. When implemented in team-based formats, gamification also improves communication and social interaction among participants. It offers opportunities to build interpersonal connections, foster mutual respect, and develop teamwork and collaboration skills while simulating real-world professional challenges [26,27,37]. By sharing reflections and experiences, this collaborative learning process contributes to developing practical competencies. It positively influences students’ perceptions of their field and future career aspirations [30,33].

The findings of this study highlight that gamification not only improves nursing students’ competencies and confidence but also has a transformative impact on their career aspirations. Through gamification, students develop a deeper understanding of the complexity of managing mental disorders, which helps them approach mental health with a more empathetic and proactive attitude. Gamification is therefore established as a valuable tool in training future nursing professionals, promoting a holistic and positive approach to mental health care that will benefit students and patients.

### Practical and Research Implications

The practical implications of this study highlight the importance of integrating gamification tools, such as interactive cards, into health sciences education, specifically in the training of nursing students. This approach improves understanding of mental disorders and fosters the development of emotional competencies and key skills, such as empathy, effective communication, and critical reflection, essential for providing comprehensive and humanized care. Incorporating these tools can also enhance students’ ability to engage with patients more compassionately and informally, improving patient outcomes. Incorporating these tools can also enhance students’ ability to engage with patients more compassionately and informally, improving patient outcomes. Likewise, the implementation of these strategies can be extended to clinical settings, where interactive tools could be adapted for patient education or used as support in therapeutic contexts. In addition, these activities promote collaborative learning, creating a space for open discussion and exchange of perspectives among students, which enhances their preparedness to address complex situations in professional practice. This approach is also aligned with the principles of experiential learning, where students learn through direct practice and reflection on their experiences, reinforcing understanding and retention of key concepts.

In terms of research, this study opens the door to future explorations that evaluate the longitudinal impact of gamified tools on understanding and empathy toward mental disorders. It would be relevant to investigate how this approach influences the development of professional competencies over time. In addition, it is suggested that studies be conducted in different cultural and educational contexts to determine the transferability of the findings and compare the effectiveness of gamification versus traditional teaching methodologies. Research should also focus on developing and customizing gamified tools adapted to the specific needs of the students and disorders studied. Finally, exploring emerging technologies, such as virtual or augmented reality, could maximize immersion and enrich student learning in this field.

### Strengths and Limitations

This study highlights 2 fundamental aspects. First, it proposes an innovative perspective on transforming the university educational model, advocating using gamification tools, such as interactive cards, to enrich understanding and reflection on mental disorders. Second, gamified strategies stimulate student interest and participation and facilitate more profound and meaningful learning.

Regarding the study’s limitations, it is relevant to mention that it was conducted with nursing students from a university in Spain, which restricts the generalizability of the findings to other contexts or institutions. This study reflects students’ perceptions of using interactive cards as a gamification tool to explore mental disorders. It would be valuable to extend qualitative research in a broader framework, incorporating a variety of gamification tools and complementing it with quantitative studies that measure the effectiveness of these innovative educational strategies.

### Conclusions

This qualitative study has shown that gamification, used as a teaching strategy, can be an effective tool to improve the understanding of mental disorders among nursing students. Participants highlighted a higher degree of empathy, motivation, and commitment to learning, as well as a better internalization of content related to mental health. The results also reveal that nursing students face difficulties when participating in gamification activities related to mental disorders, mainly due to their limited prior experience in this field. In this context, implementing graphics as a gamified resource was shown to be an effective strategy to address these difficulties. These graphics, used as metaphors, provided an interactive and reflective framework that facilitated learning, allowing students to explore their emotions less directly and express them symbolically.

This visual approach helped participants reflect on their internal conflicts and project their goals and aspirations while developing self-assessment skills by identifying emotions, conflicts, and possible solutions. In this way, gamification serves to transmit knowledge and as a catalyst for students’ personal and emotional growth in the mental health field. From a practical point of view, these findings suggest that including gamified dynamics—especially those with visual and symbolic components—in nursing training programs can facilitate a more meaningful, empathetic, and participatory approach to studying mental disorders.

As future lines of research, it is proposed to replicate this study with more extensive and more heterogeneous samples and to complement the qualitative approach with quantitative studies that evaluate the impact of gamification on variables such as academic performance, knowledge retention, or the development of emotional competencies. It would also be relevant to explore teachers’ and clinicians’ perceptions of integrating gamified methodologies in teaching content related to mental health.

## Supplementary material

10.2196/71921 Multimedia Appendix 1 Detailed descriptions of each interactive card.

## Abbreviations

COREQ: Consolidated Criteria for Reporting Qualitative Research
ED: eating disorder
FG: focus group
PD: personality disorder
