# Correction: Gamification as a Tool for Understanding Mental Disorders in Nursing Students: Qualitative Study

**DOI:** 10.2196/83237

**Published:** 2025-09-22

**Authors:** Pablo Del Pozo-Herce, Alberto Tovar-Reinoso, Eva García Carpintero-Blas, Ana Casaux Huertas, Regina Ruiz de Viñaspre-Hernández, Antonio Martínez-Sabater, Elena Chover-Sierra, Marta Rodríguez-García, Raul Juarez-Vela

**Affiliations:** 1 Research Group on Innovation in Health Care and Nursing Education (INcUidE), UNIE University, Madrid, Spain; 2 Escuela de Enfermería Fundación Jiménez Díaz – Universidad Autónoma de Madrid (FJD-UAM), Campus de Villalba, Instituto de Investigación Sanitaria Fundación Jiménez Díaz (IIS-FJD, UAM), Madrid, Spain; 3 Madrid Renal Foundation, Madrid, Spain; 4 Research Group in Care GRUPAC, Department of Nursing, Faculty of Health Sciences, Universidad de La Rioja, Logroño, Spain; 5 Care Research Group (INCLIVA), Hospital Clínico Universitario de Valencia, Valencia, Spain; 6 Nursing Care and Education Research Group (GRIECE), Nursing Department, Universitat de València, Menendez y Pelayo, 19, Valencia, 46010, Spain, 34 639871179; 7 Internal Medicine Department, Consorcio Hospital General Universitario, Valencia, Spain

In “Gamification as a Tool for Understanding Mental Disorders in Nursing Students: Qualitative Study” (JMIR Nurs 2025;8:e71921), the authors made two changes.

Under “Ethical Considerations,” the first sentence has been changed from:

*Ethical authorization to conduct the research was obtained from the Research Ethics Committee of the Instituto de Investigación Sanitaria Fundación Jiménez Díaz (CEI_PIC321-23_FJD).*

And now reads as follows:

*The study was submitted to the Research Ethics Committee of the Instituto de Investigación Sanitaria Fundación Jiménez Díaz (CEIm-FJD) for review. Although the committee determined that the project fell outside its scope as it did not constitute biomedical research, it found no ethical objections to the documentation provided.*

Additionally, the following affiliation has been added for authors Pablo del Pozo-Herce, Alberto Tovar-Reinoso, and Ana Casaux Huertas (with all following affiliations renumbered accordingly):

*Escuela de Enfermería Fundación Jiménez Díaz–Universidad Autónoma de Madrid (FJD-UAM), Campus de Villalba, Instituto de Investigación Sanitaria Fundación Jiménez Díaz (IIS-FJD, UAM), Madrid, Spain*

The corrections will appear in the online version of the paper on the JMIR Publications website, together with the publication of this correction notice. Because this was made after submission to PubMed, PubMed Central, and other full-text repositories, the corrected article has also been resubmitted to those repositories.

