# Supplementary material for: Gamification as a Tool for Understanding Mental Disorders in Nursing Students: Qualitative Study
Source: JMIR Nurs. 2025 Jun 20;8:e71921. doi: 10.2196/71921 (PMC12204240; doi:10.2196/71921)
Supplement: Multimedia Appendix 1 [file nursing-v8-e71921-s001.docx]

**Appendix 1.** Interactive cards used in the activity.

| 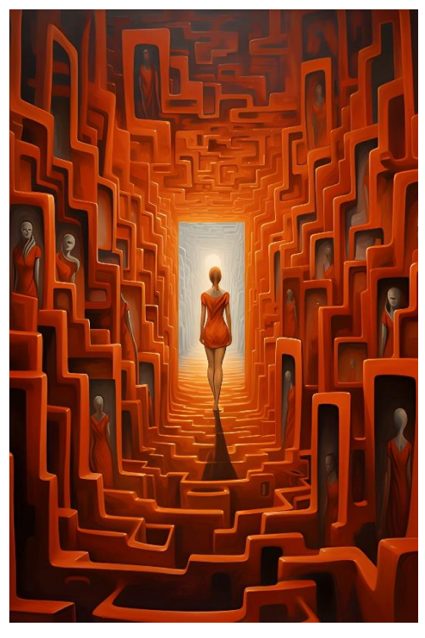  **(a): Interactive card 1** | **Description:** A central figure walks towards the light through a labyrinthine corridor formed by multiple red squares, each containing similar figures, as if they were reflections. The red tones dominate the scene, generating a sense of urgency, stress, and confusion. The silhouettes look out from their frames, all similar but nonidentical. This journey towards the light symbolizes the search for one's identity amid a distorted perception of oneself and others.  **Interpretation: This interactive card introduces the theme of personality disorders and serves to discuss how the perception of the world and the "self" is constantly changing. The labyrinth-like environment represents the multiple masks, roles, and fragmentations of identity that a person with this type of disorder may experience. The distorted mirrors remind us that we do not always see ourselves as we are or as others see us. The red reinforces the constant emotional impact and internal overload accompanying these experiences. The figure walking toward the light symbolizes the possibility of integration, awareness, and growth despite the internal chaos.** |
| --- | --- |
| 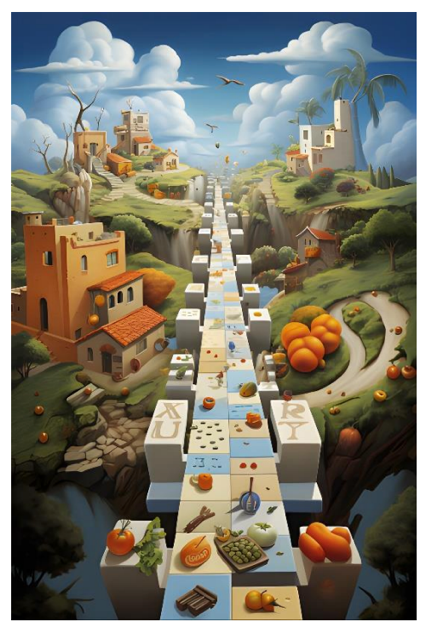  **(b): Interactive card 2** | **Description:** A tiled path runs through a fantasy landscape: houses, green hills, and blue skies that evoke a "normal" environment. However, the path is covered with food boxes and everyday items (such as fruits, sweets, and letters), transforming daily into obstacles. Each step seems carefully calculated as if the person must avoid falling into "forbidden" boxes. Life goes on naturally on the sides, but the person walking on the board lives a different reality.  **Interpretation: This interactive card represents the inner experience of a person with an eating disorder. What for others is an ordinary situation (a birthday, a walk, a meal) for this person becomes a constant game of mental survival. Every event becomes a challenge, a trap, or a threat. The card also shows a beautiful and promising landscape beyond the board, alluding to the life that awaits after recovery, even if it cannot yet be seen clearly. It serves to work the invisibility of suffering and hope: even if the road is difficult, there is life beyond the disorder.** |
| 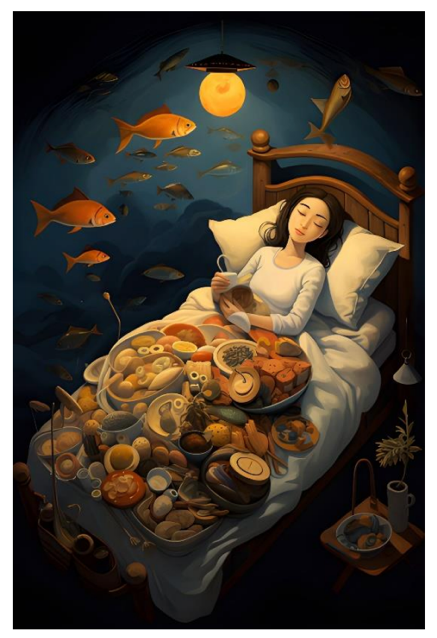  **(c): Interactive card 3** | **Description**: The card presents a night scene with a person in bed, apparently restless or unable to sleep. Around it, elements that reinforce the feeling of obsession and anguish may appear: shadows in the room, clocks marking the passage of time, floating thoughts with images of food, or words related to guilt and desire.  **Interpretation: The interactive card symbolizes the constant presence of food in the minds of those with an ED, even during rest. Many people with these disorders dream about food or experience it as an obsession that prevents them from sleeping. This image not only reflects the psychological impact of the ED but also the emotional suffering involved in the internal struggle between desire and restriction.** |
| 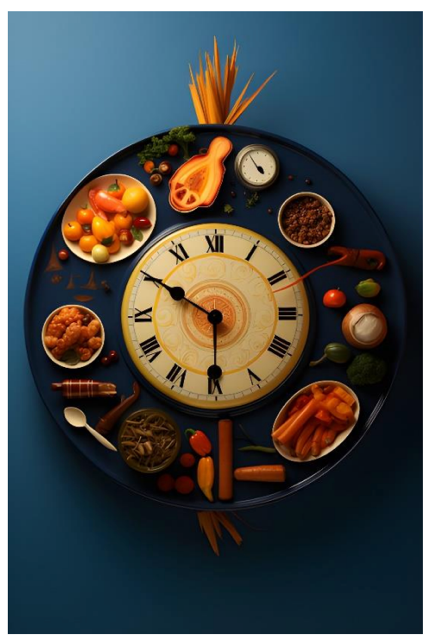  **(d): Interactive card 4** | **Description: The interactive card shows a variety of foods arranged in an environment that suggests order or chaos. Some elements may represent strict schedules, such as clocks or calendars, or, on the contrary, a disorderly distribution of food. Symbols that reinforce the idea of control and self-censorship may also appear, such as locks, rules, or notes with restrictions.**  **Interpretation: This card represents two extremes of the relationship to eating in ED. On the one hand, extreme rigidity is when the person only eats when "it's time" and represses any feeling of hunger outside those moments. On the other, a total lack of control is characterized by a lack of routines and impulsive eating episodes. Both scenarios reflect an internal struggle with eating, where the pleasure of eating is replaced by guilt or anxiety.** |
